# 3D-Printed Gentamicin-Releasing Poly-ε-Caprolactone Composite Prevents Fracture-Related *Staphylococcus aureus* Infection in Mice

**DOI:** 10.3390/pharmaceutics14071363

**Published:** 2022-06-28

**Authors:** Clara Guarch-Pérez, Bahaa Shaqour, Martijn Riool, Bart Verleije, Koen Beyers, Chris Vervaet, Paul Cos, Sebastian A. J. Zaat

**Affiliations:** 1 Department of Medical Microbiology and Infection Prevention, Amsterdam Institute for Infection and Immunity, Amsterdam UMC, University of Amsterdam, Meibergdreef 9, 1105 AZ Amsterdam, The Netherlands; c.m.guarchperez@amsterdamumc.nl (C.G.-P.); s.a.zaat@amsterdamumc.nl (S.A.J.Z.); 2 Mechanical and Mechatronics Engineering Department, Faculty of Engineering & Information Technology, An-Najah National University, Nablus P.O. Box 7, Palestine; bahaa.shaqour@gmail.com; 3 Laboratory for Microbiology, Parasitology and Hygiene (LMPH), Faculty of Pharmaceutical, Biomedical and Veterinary Sciences, University of Antwerp, Universiteitsplein 1, 2610 Antwerp, Belgium; paul.cos@uantwerpen.be; 4 Voxdale bv, Bijkhoevelaan 32, 2110 Wijnegem, Belgium; bart@voxdale.be (B.V.); koen@voxdale.be (K.B.); 5 Laboratory of Pharmaceutical Technology, Department of Pharmaceutics, Ghent University, Ottergemsesteenweg 460, 9000 Ghent, Belgium; chris.vervaet@ugent.be

**Keywords:** 3D printing, *Staphylococcus aureus*, fused filament fabrication, bone-related infection, mouse model

## Abstract

Bacterial infections are a serious healthcare complication in orthopedic and trauma surgery worldwide. Compared to systemic, local antibiotic prophylaxis has been shown to provide a higher antibiotic dose and bioavailability at the bone site with minimum toxic effects. However, there are still not enough biomaterial and antibiotic combinations available for personalized implant sizes for patients. The aim of this study was to develop a bone fixation plate coating made of a composite of poly-ε-caprolactone, hydroxyapatite and halloysite nanotubes loaded with gentamicin sulphate and fabricated via fused filament fabrication 3D printing technology. The mechanical and thermal properties of the biomaterial were analyzed. The in vitro release kinetics of gentamicin sulphate were evaluated for 14 days showing a burst release during the first two days that was followed by a sustained release of bactericidal concentrations. The composite loaded with 2 and 5% gentamicin sulphate exhibited complete antimicrobial killing of *Staphylococcus aureus* in an ex vivo mouse femur fixation plate infection model. Moreover, a fixation plate of the composite loaded with 5% of gentamicin sulphate was able to prevent *S. aureus* infection in the bone and surrounding tissue in an in vivo mouse bone fixation plate infection model 3 days post-surgery. In conclusion, the newly developed composite material successfully prevented infection in vivo. Additionally, the ability to use fused filament fabrication 3D printing to produce patient-specific implants may provide a wider range of personalized solutions for patients.

## 1. Introduction

Bacterial infections are a serious healthcare complication in orthopedic and trauma surgery worldwide [1]. The bacterial species *Staphylococcus aureus* and *Staphylococcus epidermidis* are the most common pathogens associated with orthopedic infections [2,3]. Fractures have infection rates ranging from 5 to 10% for closed fractures and even up to 30% for open fractures [1]. These infections are difficult to treat due to the ability of sessile bacteria in biofilms to evade the immune response and antibiotic treatment [4]. Clinically, implant-associated infections are mainly prevented by skin antiseptics and systemic antibiotic prophylaxis. Despite the reduction in infection rates, systemic antibiotic prophylaxis cannot provide sufficient protection in the tissue surrounding the medical device because of a limited antibiotic concentration in the bone [3]. Local antibiotic prophylaxis has been shown to provide a higher antibiotic dose and bioavailability at the bone site with minimum toxic effects [5,6].

Polymeric carriers have been extensively studied for the delivery of antibiotics. Poly-ε-caprolactone (PCL) is commonly used in drug delivery medical applications [7,8]. PCL was selected in our study because of its low melting temperature, biocompatibility, drug release characteristics and biodegradability [9]. The low processing temperature allows the incorporation of thermally labile drugs to produce new formulations with certain functionalities, such as antimicrobial implantable devices. However, PCL has poor mechanical properties, thus, many researchers avoid using this polymer for load-bearing applications. Alternatively, PCL can be used along with load-bearing material as a layer for controlling the drug release or as a drug-carrying coating. Bose et al., used PCL as a coating for minimizing the burst release of alendronate from the surface of titanium disks mimicking implantable devices [10]. Moreover, Jahanmard et al., produced an antibiotic-loaded PCL coating for titanium implants via electrospinning [11]. To improve the biological and mechanical properties of PCL, hydroxyapatite (HA) and halloysite nanotubes (HNT) have been incorporated [12].

Three-dimensional printing technology has been extensively used for producing drug delivery systems using various polymers [7,8]. Moreover, multi-material 3D printing can combine two or more materials with different characteristics in one structure. Several studies have investigated the compatibility and performance of printing using two materials. Such an approach may provide the advantages of using non-load bearing material (i.e., PCL) with a load-bearing material (i.e., polylactic acid (PLA) [13,14]. In such systems, the load-bearing material may be used as a core material for its good mechanical properties, while the non-load-bearing material can be used as a shell for its capabilities as a drug carrier and releasing matrix.

In our study, we focused on the fabrication of a 3D-printed PCL composite to release gentamicin sulphate (GS) ultimately intended to be a release coating on weight-bearing materials such as PLA. The composite consisted of PCL, HA and HNT, was loaded with GS and fabricated by fused filament fabrication (FFF) 3D printing technology in the shape of a bone fixation plate for its application to a mouse femur. Although not an actual bone fixation plate, we will for simplicity refer to these devices as “fixation plates” in this report. GS was chosen for its broad-spectrum antibacterial activity and thermostability required in the printing process. The implants were evaluated in in vitro, ex vivo and in vivo experiments for their antimicrobial efficacy. Our formulation of PCL-HA-HNT can be used as a coating (outer layer) during the 3D printing of implantable devices for applications such as fracture fixation. This will lay the ground for further studies aiming at using multi-material 3D printing technology to produce load-bearing implantable devices with specific drug release properties.

## 2. Materials and Methods

### 2.1. Preparation of Filaments

**Figure 1:**
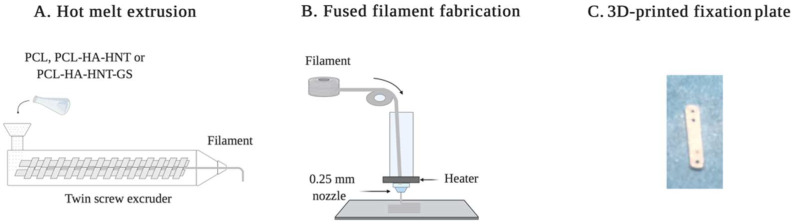
Schematic representation of (**A**) the hot melting extrusion process and (**B**) the fused filament fabrication printing process. (**C**) Picture of a 3D-printed fixation plate by fused filament fabrication.

PCL in powder form, Capa™ 6506 was kindly provided by Perstorp (Warrington, UK). Capa™ 6506 is a PCL grade with a molecular weight of 50,000 Da [15]. HNT (CAS No. 1332-58-7) and HA (CAS No. 1306-06-5) were purchased from Sigma-Aldrich (Burlington, MA, USA). HA and HNT were added to PCL at 20% and 2.5% (*w/w*), respectively, with a batch size of 40 g. The mixture was then mixed using a mortar and pestle manually. GS in powder form (Sigma-Aldrich) was added to two 40 g PCL-HA-HNT composite batches at either 2% or 5% (*w/w*) and then mixed with mortar and pestle. Pure PCL, non-loaded and GS-loaded PCL-HA-HNT composites were hot-melt extruded using a mini extruder (Minilab II, Haake Thermo Fisher Scientific, Dreieich, Germany) equipped with a co-rotating screws system (Figure 1). The extrusion temperature for the neat PCL was set to 65 °C while for the composites the temperature was set to 75 °C. The extrusion speed was kept constant at 30 rpm and the mixture was manually fed. The torque on the motors was in the range between 40 and 50 N.cm. The extruder was fitted with a diameter of 2 mm. A filament pulling system (developed in-house) was attached to the extruder with a cooling fan and a pulling speed ranging between 300 and 500 m/min to assure the production of a filament with a constant diameter.

### 2.2. Filament Fused Fabrication (3D Printing)

A 3D model of a fixation plate for a mouse femur was designed using Creo PTC parametric (v.5.0, PTC, Boston, MA, USA) with a length, width and thickness of 8.5, 1.5 and 0.7 mm, respectively. Afterwards, the model was sliced using PrusaSlicer (v.2.1, Prusa Research, Prague, Czech Republic) and a G-code file was generated. The produced file was uploaded to a Prusa i3 MK3S FFF 3D printer (Prusa Research) equipped with a 0.25 mm nozzle. The nozzle’s temperature for printing PCL, non-loaded and GS-loaded PCL-HA-HNT was 120 °C, 130 °C, and 180 °C, respectively. This was due to the smaller nozzle diameter and the need for reducing the melt viscosity which required an even higher temperature after adding GS. The layer height selected was 0.2 mm. A rectilinear 100% infill was chosen. The printing speed was set between 25 and 45 mm/s and the bed temperature was 30 °C.

### 2.3. Thermal Analysis

The produced filaments were analyzed by Differential Scanning Calorimetry (DSC; Q2000 TA instruments, Leatherhead, UK) to investigate the crystallinity of PCL. Tzero pans (TA instruments) were filled with approximately 5–10 mg of sample and placed in the DSC instrument after being non-hermetically sealed with Tzero lids using a Tzero Press (TA instruments). An empty Tzero pan was used as a reference. A heating run at a rate of 10 °C/min from 0 to 100 °C was performed. The DSC instrument was equipped with a refrigerated cooling system and dry nitrogen at a flow rate of 50 mL/min. The percentage of PCL crystallinity was calculated based on the following equation using the melt enthalpy obtained in the DSC experiments:

$$Crystallinity=\frac{H_{s}}{H_{fc}\times R}\times 100\%$$

$H_{s}$ is the melting enthalpy of a sample
R is the PCL content in a sample
$H_{fc}$ is the melting enthalpy of 100% crystalline PCL which is 142 J/g [9]

### 2.4. Mechanical Testing

The mechanical properties of the produced filaments were tested using tensile and three-point bending tests (*n* = 5) [16]. A texture analyzer (TA.HDplusC, Stable Micro Systems, Surrey, UK) equipped with a 50 kg load cell was used. In the case of the tensile testing, pneumatic grips were fitted on the machine. Samples with a length of 80 mm with a gauge distance of 60 mm were used. In order to fulfil the ISO-527 standard criteria, a testing speed of 0.6 mm/s was selected which represents a 1%/min of the initial length of the sample. The elastic modulus was calculated between 0.5% and 2% strain. Furthermore, the three-point bending test was conducted using a rig that has two supports on the bottom and a central moving part on the top. The gap between the two supports was 8 mm, and the speed of the moving top support was 1.2 mm/min. A total displacement of 2 mm was allowed during the test. The test samples were cut into segments of 20 mm in length. The flexural stress and strain were calculated based on the following equations, and the flexural elastic modulus was calculated between 1% and 5% strain:

$$\sigma_{f}=\frac{FL}{\pi R^{3}}$$

$\sigma_{f}$ is the flexural stress (MPa)
F is the applied force (N)
L is the gap (mm)
R is the radius of the specimen (mm)

$$\varepsilon_{f}=\frac{600sh}{L^{2}}$$

$\varepsilon_{f}$ is the flexural strain (%)
s is the deflection (mm)
h is the thickness of the test specimen (mm)
L is the gap (mm)

### 2.5. Bacterial Strain and Inoculum Preparation

*S. aureus* JAR 06.01.31, a strain originally isolated from an infected hip prosthesis (University Hospital Basel, Basel, Switzerland, [17]), was used. The inoculum preparation for the ex vivo and in vivo bone infection study consisted of the resuspension of a single *S. aureus* colony in 5 mL of tryptic soy broth (TSB; Oxoid, Basingstoke, UK) and incubation for 3 h at 37 °C and 120 rpm. Next, the culture was centrifuged at 8000× *g* for 5 min and resuspended in 0.9% of NaCl (saline; Fresenius Kabi, Zeist, The Netherlands) at a concentration of 1 × 10^7^ colony forming units (CFU)/mL.

### 2.6. Thermal Stability of Gentamicin Sulphate

A minimum inhibitory concentration (MIC) assay was used to determine the effect of heating on the stability of GS. *S. aureus* JAR 06.01.31 was cultured to the mid-logarithmic growth phase in TSB at 37 °C and 120 rpm to an absorbance of 0.03, corresponding to a concentration of 1 × 10^7^ CFU/mL. Ten microliters of this bacterial suspension were added to 90 µL of TSB with either untreated GS (control), or GS heated at 180 °C serially diluted in TSB from 128 µg/mL to 0.125 µg/mL in flat-bottom microtiter plates (Greiner bio-one, Kremsmünster, Austria) and incubated in a box in a humid atmosphere, overnight at 37 °C and rotating at 120 rpm. The MIC, which is the lowest concentration of an antimicrobial agent to prevent visible growth, was determined by visual assessment. The minimum bactericidal concentration (MBC) was assessed by plating duplicate 10 µL aliquots from the wells without visible growth and from the well with the lowest GS concentration with visible growth on blood agar plates (Oxoid, Basingstoke, UK) which were incubated at 37 °C overnight.

### 2.7. Cell Viability Assay

A cell viability assay was performed to investigate any possible change in the cytotoxic effect of GS due to heating. The L929 mouse fibroblasts cell line was purchased from the European Collection of Authenticated Cell Cultures (ECACC, Salisbury, UK) and cultured in Dulbecco’s modified Eagle’s medium (DMEM; Thermofisher, Walthman, MA, USA) supplemented with 10% of fetal bovine serum (FBS; Thermofisher) and 1% penicillin and streptomycin (pen/strep) in cell culture dishes. Cells were incubated at 37 °C, 5% CO_2_ and 99% relative humidity. Cells were detached with 1× trypsin-Ethylenediaminetetraacetic acid (EDTA; 0.25% trypsin, 1 mM EDTA Na, Thermofisher) and counted. One hundred microliters with 10^3^ cells were seeded per well in a 96-well cell culture plate and incubated for 24 h at 37 °C and 5% CO_2_. The next day, the medium was removed and 200 µL of GS or GS heated both at 10 µg/mL in DMEM were added to the wells of three plates. Dimethyl sulfoxide (DMSO; Sigma-Aldrich) at 10% in DMEM was used as a positive control for cytotoxicity and DMEM with pen/strep was used as a negative control. The cells were incubated for 24, 48 or 72 h at 37 °C in 5% CO_2_. At each time point, the medium was removed, and cells were washed with 200 µL of 1x D-phosphate buffered saline (D-PBS; Thermofisher). To confirm the number of viable cells, 100 µL of DMEM and 20 µL of the reagent of Cell Titer 96 Aqueous One Solution Cell Proliferation Assay (Promega, Madison, WI, USA) were added per well and incubated for 2 h at 37 °C and then the absorbance was measured at 490 nm with a spectrophotometer (Synergy H1, Biotek, Winooski, VT, USA).

### 2.8. Gentamicin Sulphate Release

PCL-HA-HNT fixation plates loaded with 2 and 5% of GS were placed in triplicates in Eppendorf tubes with 500 µL of PBS (Merck, Horsham, PA, USA) at 37 °C and 120 rpm. The plates were transferred to new tubes with fresh PBS after 1, 6 and 24 h, and 2, 3, 4, 6, 8, 11 and 14 days. GS release from PCL-HA-HNT fixation plates was determined in vitro by combining 130 µL of the eluates with 30 µL of o-phthalaldehyde reagent (Sigma-Aldrich) and 140 µL of isopropanol and measuring the fluorescence emission at 455 nm after excitation at 340 nm of excitation, with a fluorimeter (Synergy H1, Biotek). A calibration curve was plotted for GS to determine the concentration of the drug released from the tubes. This curve ranged from 1 to 50 µg/mL with R2 equal to 0.9899. The cumulative drug release was calculated based on the total loading amount (2 and 5% *w/w*) present in the fixation plates.

### 2.9. Adhesion Assay

An in vitro bacterial adhesion assay was performed to evaluate the antimicrobial efficacy of the fixation plates loaded with 2 and 5% of GS to prevent bacterial growth on the fixation plates. In addition, planktonic bacterial growth in the surrounding medium was assessed. *S. aureus* was cultured to the mid-logarithmic growth phase in TSB at 37 °C and 120 rpm and diluted in TSB to 1 × 10^6^ CFU/mL. The fixation plates were incubated in 0.5 mL of this suspension in 1.5 mL Eppendorf tubes overnight at 37 °C shaking at 120 rpm. The fixation plates were washed twice with demi water, transferred to 1.5 mL Eppendorf tubes with 0.5 mL of PBS, vortexed for 30 s and sonicated at 35 kHz for 15 min in a water bath sonicator (Elma Transsonic T460, Elma, Singen, Germany). This procedure does not affect the viability of the bacteria but releases them from the surface [18]. Moreover, the planktonic bacterial growth in the medium was collected. The sonicates and medium collected were ten-fold serially diluted and the number of viable bacteria was determined by quantitative culture on blood agar plates.

### 2.10. Scanning Electron Microscopy

Bacterial attachment to the fixation plates was studied by scanning electron microscopy (SEM) of the plates from the adhesion assay (see above). The fixation plates were fixed in a solution of 4% (*v/v*) paraformaldehyde with 1% (*v/v*) glutaraldehyde (both Merck, USA) overnight at room temperature. The fixation plates were rinsed twice with demineralized water for 10 min and dehydrated in a graded ethanol concentration series from 50% to 100% of ethanol. The fixation plates were immersed in hexamethyldisilane (Polysciences Inc., Warrington, FL, USA) overnight. Before imaging, samples were mounted on aluminum SEM stubs and sputter-coated with a 4 nm platinum–palladium layer using a Leica EM ACE600 sputter coater (Microsystems, Wetzlar, Germany). Images were acquired at 3 kV using a Zeiss Sigma 300 SEM (Zeiss, Oberkochen, Germany) at the Electron Microscopy Center Amsterdam (ECMA; Amsterdam UMC, Amsterdam, The Netherlands). Of each plate, 4 fields were inspected and photographed at magnifications of 100×, 500× and 1000×.

### 2.11. Ex Vivo Mouse Experiment

The antimicrobial activity of the non-loaded and 2 or 5% (*w/w*) GS-loaded PCL-HA-NT fixation plates (*n =* 6) was evaluated in an ex vivo model utilizing explanted femurs from mouse cadavers, provided by the Animal Research Institute AMC (ARIA) of the Amsterdam UMC (Amsterdam, The Netherlands). Firstly, the skin of the legs was disinfected with 70% ethanol and subsequently removed, followed by collecting the femurs. Then, the fixation plate was placed on a femur, and two holes were drilled with a drill bit of 0.3 mm from RISsystem (Landquart, Switzerland) in the bone and mounted with two titanium pins. Once the fixation plate was mounted, a hole of 0.5 mm was drilled in the center of the femur and 2.5 µL of the *S. aureus* JAR 06.01.31 inoculum (containing 10^4^ CFU) was pipetted into the hole.

**Figure 2:**
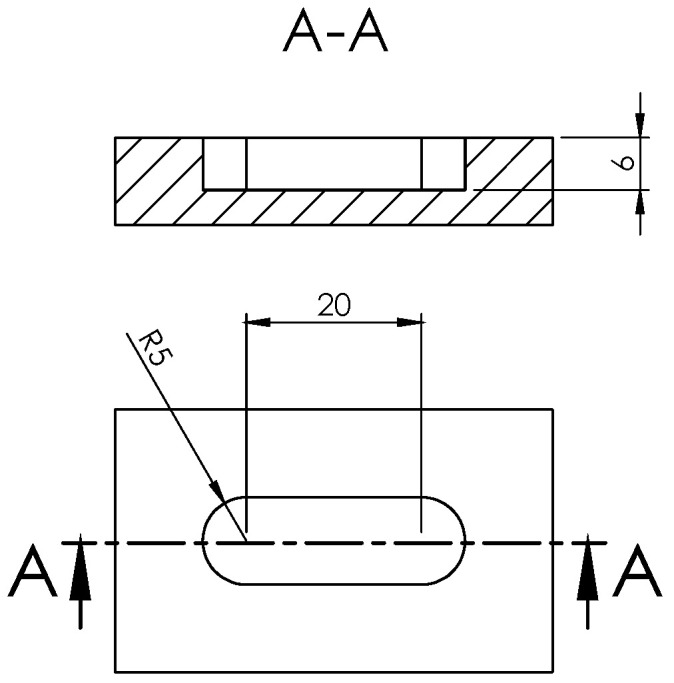
Schematic dimensions in mm of the self-designed wells for the ex vivo experiment.

Then, each mouse femur with the infected fixation plate was placed individually in wells self-designed as described in Figure 2. To mimic the surrounding tissue, low melting agarose (Sigma-Aldrich) was added to each well, covering the femur and fixation plate. One extra group (*n* = 6) of femurs without fixation plate nor inoculum was included as a control for contamination.

Once the agarose solidified, the entire system was incubated at 37 °C for 24 h. The next day, the femurs were extracted from the agarose, separated from the plates and submerged in 500 μL of PBS with 0.5% Tween 80 (Thermofisher) and 15 zirconia beads (Ø 2 mm, BioSpec, USA, [19]). The agarose and fixation plates were separately placed in tubes with 500 μL of PBS. The bones were homogenized using a MagNA Lyser (Roche Diagnostics, Basel, Switzerland) at 7000 rpm for 2 cycles of 30 s each, with cooling on ice for 30 s in between. The fixation plates were vortexed for 30 s and sonicated in a water bath sonicator (Elma Transsonic T460, Elma) for 15 min. The resulting homogenates and sonicates were quantitatively cultured on blood agar plates at 37 °C overnight.

### 2.12. Animals

The study was approved by the animal ethical committee of the Amsterdam UMC (DMB19-8484-2-02; 30 June 2021). Twelve specific-pathogen-free skeletally mature, approximately 6 months old, female C57BL/6/JRccHsd mice (Envigo, Horst, The Netherlands) were used in this study (*n* = 6 per group). One group received the non-loaded PCL-HA-HNT bone fixation plate and the second group received the PCL-HA-HNT loaded with 5% of GS bone fixation plate.

### 2.13. In Vivo Bone Infection Mouse Model

**Figure 3:**
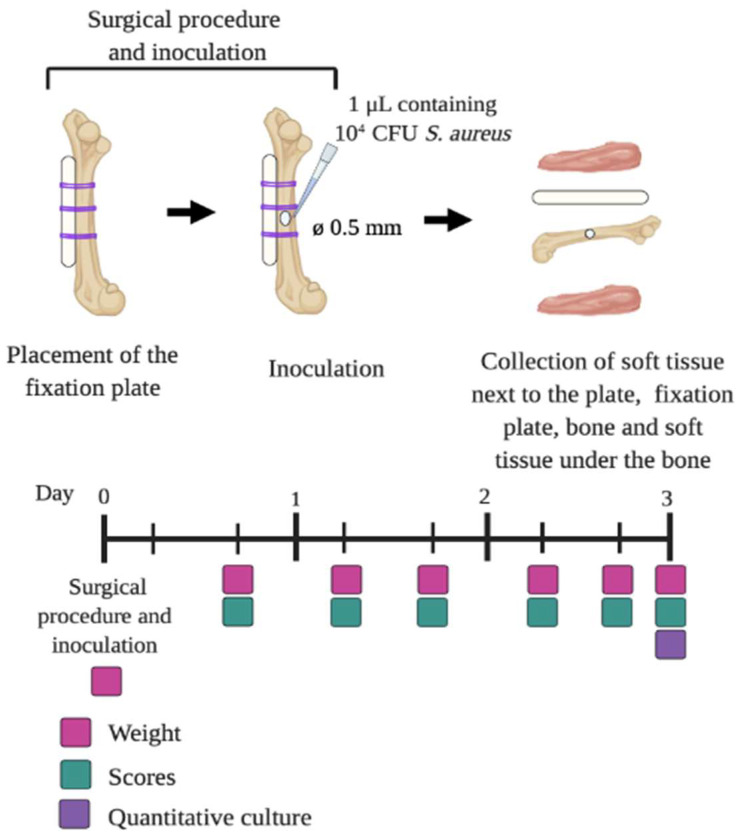
Schematic representation of the in vivo surgical procedure.

At least 30 min prior to the surgical procedure and at 6 h and 24 h post-surgery, the mice received a subcutaneous injection of buprenorphine (0.1 mg/kg; Temgesic, RB Pharmaceutical Limited, Hull, UK) for pain control. The mice were anaesthetized with 2% isoflurane (Pharmachemie, Haarlem, The Netherlands) in O_2_, placed in a prone position under the microscope (Microsystems) and their left leg was shaved and sterilized with 70% ethanol. Then, a longitudinal skin incision was made along the femur. The subcutaneous fascia lata was cut and the muscles around the bone were split with tweezers to create space around the bone. Then, either a non-loaded or 5% (*w/w*) GS-loaded PCL-HA-HNT fixation plate was placed on the femur and fixed with three individual 6-0 vicryl sutures (Ethicon, Raritan, NJ, USA) around the bone (Figure 3). The use of the sutures to fix the plate to the bone was necessary because of the fragility of the plates, which did not allow the fixation of the plates with screws. Once the plate was in place, a hole was drilled in the center of the femur until the medullar cavity was reached, using a drill bit of 0.5 mm (RISystem). A 1 µL inoculum containing 10^4^ CFU of *S. aureus* JAR 06.01.31 was pipetted into this hole. Then, the facia lata and the skin were closed with 6-0 vicryl sutures.

Caprofen (0.6 mg/mL; Rimadyl, Zoetis, Capelle aan den Ijssel, The Netherlands) was administered to the drinking water for the following 3 days for pain control. Animals were monitored twice daily for 3 days using a scoring system that included general behavior (posture and facial expression), external appearance (fur, eyes, skin), movement, feces and urine, breathing, wound status, and grimace score and weight (scale 0–21, Table 1, [20,21]. According to the scoring system, the mice would be euthanized in the case of the score being higher than 10, or when the weight loss is >15% compared to the highest body weight. After 3 days post-surgery, the animals were euthanized using CO_2_. The left leg operated femur, the fixation plate and the tissue on top of the plate and the tissue below the femur were harvested separately in sterile containers with 500 µL of PBS with 0.5% of Tween 80. The right bone and soft tissue from the right non-operated leg were also collected and placed in sterile containers with 500 µL of PBS with 0.5% of Tween 80 to evaluate possible hematogenous dissemination. The containers for the bone and the tissue also contained zirconia beads (15 beads for bone and 5 beads for tissue). The soft tissue and the bone were homogenized with the MagNA Lyser system (Roche) for 3 cycles at 7000 rpm for 30 s with cooling on ice between cycles. The fixation plates were washed once with 0.5 mL of PBS, vortexed and sonicated for 15 min. Bacteria were quantified by culturing serial dilutions on blood agar plates which were incubated at 37 °C overnight.

### 2.14. Statistical Analysis

Statistical analysis was performed with GraphPad Prism 9 (GraphPad, San Diego, CA, USA). The statistical analysis of the in vitro and ex vivo experiments was performed using a one-way analysis of variance (ANOVA) with Dunnet’s comparison test to evaluate post-hoc differences between the groups compared to the control group. The Mann-Whitney rank sum test was used to analyze the quantitative CFU data in vivo and the differences in the total scores. For all tests, *p* ≤ 0.05 was considered significant. * Indicates a *p*-value of 0.01 to 0.05, ** indicates a *p*-value of 0.001 to 0.01, *** indicates a *p*-value of 0.0001 to 0.001, **** indicates a *p*-value < 0.0001.

## 3. Results

### 3.1. Filament Preparation and 3D Printing of Fixation Plates

Filaments of PCL, PCL-HA-HNT and GS-loaded PCL-HA-HNT were successfully produced with a diameter of around 1.75 mm. This diameter is key to allowing successful 3D printing using an FFF printer. Compared to extruding PCL, an increase of 10 degrees was needed during the extrusion of non-loaded and GS-loaded PCL-HA-HNT composites in order to reduce the melt viscosity and allow proper extrusion through the die. Moreover, the speed of the pulling system was crucial to overcoming any flow variation between batches which was caused by adding HA and HNT and also to reduce the extrudate diameter from 2 mm (die diameter) to the required filament diameter. Afterwards, the 3D printing of the plates was conducted using a 0.25 mm nozzle. However, it was observed that a higher temperature was needed for the composites. The printing temperature for PCL, PCL-HA-HNT and GS-loaded PCL-HA-HNT were 120 °C 130 °C and 180 °C, respectively.

### 3.2. Thermal Analysis

**Figure 4:**
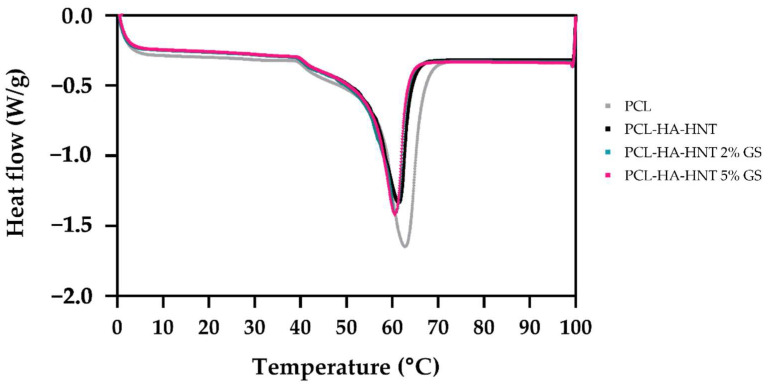
DSC analysis of produced PCL, PCL-HA-HNT and GS loaded PCL-HA-HNT at 2% and 5% filaments.

GS, HA and HNT do not show melting behavior between 0 and 100 °C [9,22]. Moreover, PCL, GS, HA and HNT are thermally stable at all printing temperatures used within this study [9,22]. Thus, DSC analysis (Figure 4) was performed to study the effect of the addition of HA, HNT and GS on the crystallinity of PCL. Table 2 shows the melting temperature of the produced filaments. It can be seen that the melting temperature (T_m_) slightly decreases after adding HA, HNT and GS. The crystallinity was calculated in the first heating cycle, with the crystallinity of PCL slightly decreased after adding the components.

### 3.3. Mechanical Testing

**Figure 5:**
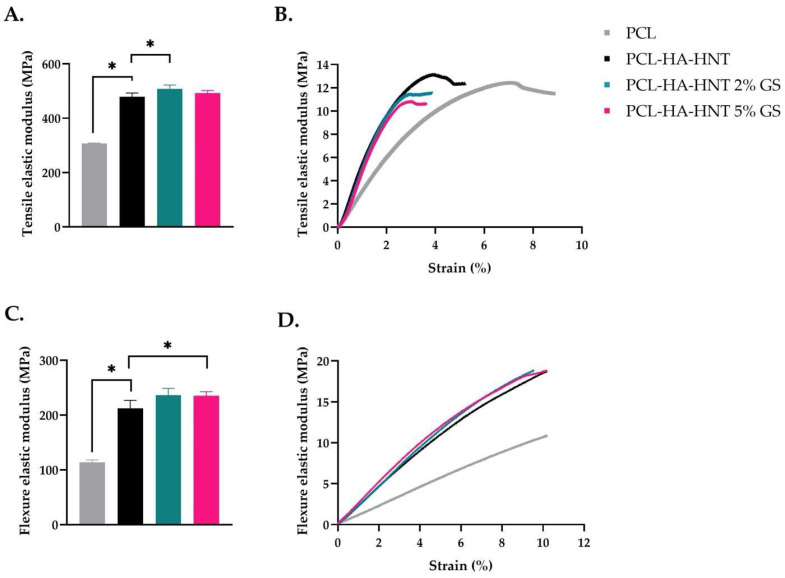
Mechanical properties of PCL, non-loaded PCL-HA-HNT and 2 and 5% GS-loaded PCL-HA-HNT. Elastic modulus and stress–strain curves for tensile (**A**,**B**) and three-point bending (**C**,**D**) tests (*n* = 5). * Indicates a *p*-value of 0.01 to 0.05.

The mechanical properties of the produced filaments were tested using tensile and three-point bending tests. The tensile elastic modulus was 307.16 ± 1.53, 479.22 ± 13.62, 508.28 ± 13.78 and 492.43 ± 39.16 for PCL, PCL-HA-HNT, 2% GS loaded PCL-HA-HNT and 5% GS loaded PCL-HA-HNT, respectively. Moreover, the bending elastic modulus was 114.10 ± 4.37, 210.69 ± 14.76, 232.33 ± 12.50 and 232.83 ± 7.14 for PCL, PCL-HA-HNT, 2% GS loaded PCL-HA-HNT and 5% GS loaded PCL-HA-HNT, respectively. The tensile and flexural elastic moduli were significantly improved after adding the HA and HNT (Figure 5A,C, respectively). The effect of GS loading on PCL composite showed a slight increase in both the tensile and flexure module. However, this change was very small when compared to the change after adding HA and HNT to PCL. The resulting stress-strain curves for the tensile and three-point bending tests can be seen in Figure 5B,D, respectively.

### 3.4. Thermal Stability of Gentamicin Sulphate

**Figure 6:**
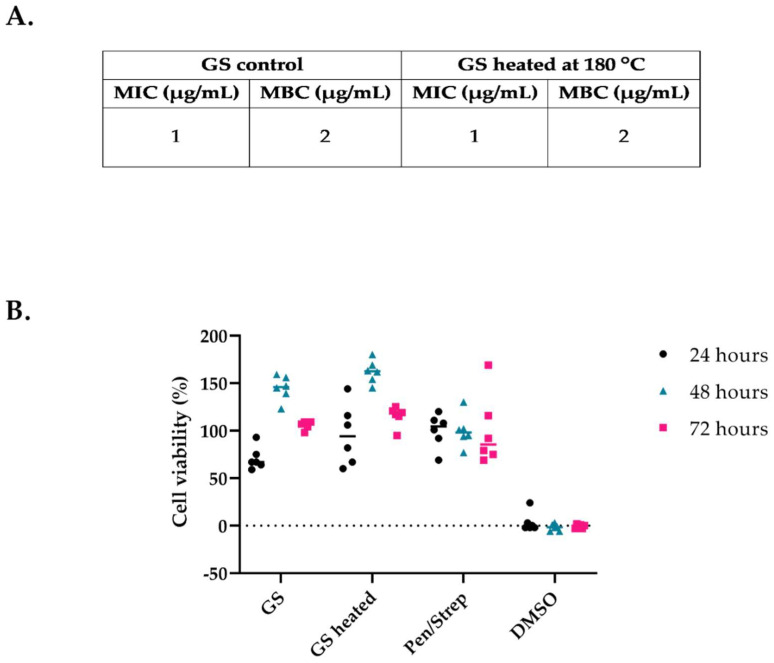
MIC/MBC of non-heated GS and GS heated at 180 °C for *S. aureus* JAR 06.01.31 (**A**). Percentage of L929 mouse fibroblast cell survival after 24, 48 and 72 h exposure to non-heated GS, heated GS, DMEM medium with Pen/strep as a negative and DMSO (10%) as a positive control for cytotoxicity (**B**). Note: DMEM medium with Pen/Strep is set at 100% cell survival (*n* = 6).

GS heated at 180 °C and non-heated GS had the same MIC and MBC values, indicating that GS is thermally stable at 180 °C and should not lose its antimicrobial activity during the printing process at 180 °C (Figure 6A). Moreover, the influence on the cell viability of GS after heating was evaluated with the L929 mouse fibroblast cell line. We confirmed that the heating process does not generate cytotoxic compounds due to any potential degradation of GS, since the activity of the cells exposed to non-treated and heat-treated GS was similar, ranging from 85 to 150%, indicating that the cells were viable (Figure 6B).

### 3.5. Gentamicin Sulphate Release from the Plate

**Figure 7:**
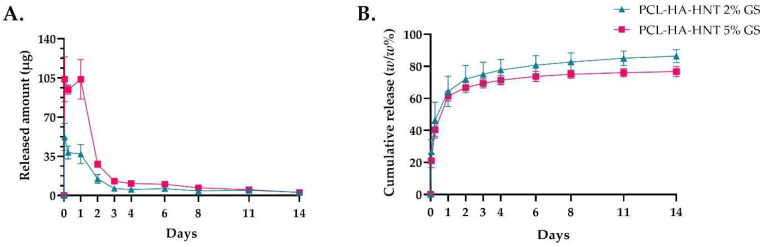
Released amount of GS (in μg) (**A**) and cumulative release (*w/w*%) (**B**) from the PCL-HA-HNT 2% GS and 5% GS fixation plates after 1, 6 and 24 h and 2, 3, 4, 6, 8, 11 and 14 days. *n* = 3 replicates per group.

PCL-HA-HNT fixation plate loaded with 2% (*w/w*) of GS showed a burst release during the first day, with 52.31 μg, 38.44 μg and 37 μg after 1, 6 and 24 h, respectively, that was followed by a sustained release for the next 14 days (ranging from 14.78 μg to 2.83 μg), likely resulting in an in vivo antimicrobial concentration above the MBC value of 2 μg/mL. PCL-HA-HNT fixation plate loaded with 5% (*w/w*) of GS showed similar release kinetics, with a burst release during the first day of 103.89 μg, 94.64 μg and 103.75 μg after 1, 6 and 24 h, respectively, that was followed by a sustained released for the next 14 days (ranging from 27.98 μg to 2.74 μg at the last day). A total of 171.86 μg (86%) and 378.62 μg (76%) were released from the 2 and 5% (*w/w*) GS, respectively (Figure 7).

### 3.6. Adhesion Assay and SEM

**Figure 8:**
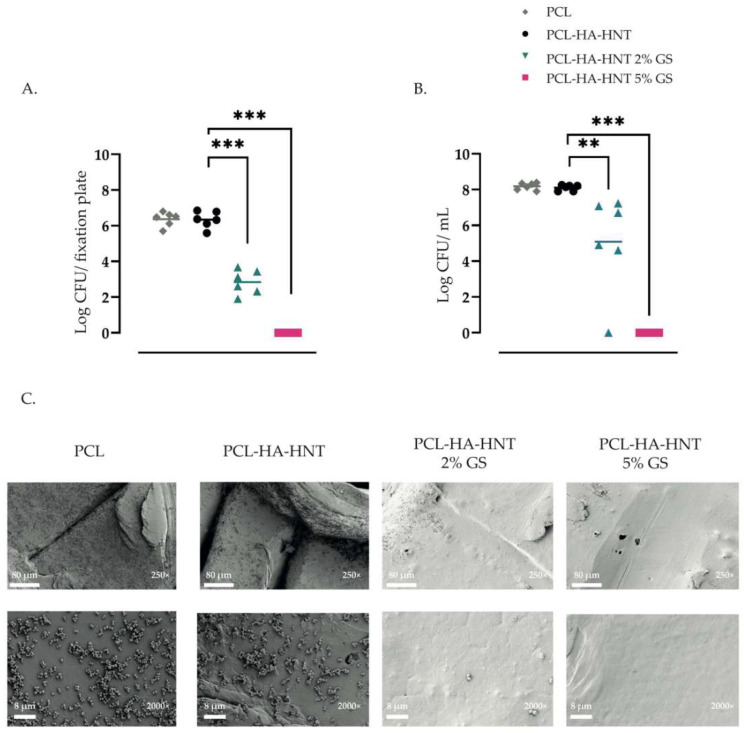
In vitro antimicrobial activity of PCL, PCL-HA-HNT, PCL-HA-HNT 2% GS and PCL-HA-HNT 5% GS fixation plates, as characterized by numbers of attached *S. aureus* CFU cultured from the plates (**A**), and bacterial growth in the medium (**B**) after inoculation with 10^6^ CFU/mL, and by visualization of bacteria attached to the surface of the fixation plates by SEM (**C**). *n* = 6 replicates per group. ** Indicates a *p*-value of 0.001 to 0.01, *** *p*-value of 0.0001 to 0.001.

The fixation plates loaded with 2% of GS significantly reduced the attachment of *S. aureus* to the plates after 24 h incubation with an average log number of CFU of 2.84 ± 0.68 (*p*-value of 0.0022), compared to the non-loaded PCL and PCL-HA-HNT plates that had an average log number of CFU of 6.37 ± 0.39 (*p*-value of 0.0022) and 6.34 ± 0.46 (*p*-value of 0.0022), respectively. Moreover, there was a significant reduction in the number of CFU present in the medium suspension of the fixation plates loaded with 2% of GS of an average log CFU/mL of 5.08 ± 2.73 compared to the suspensions of the PCL and PCL-HA-HNT fixation plates, that had an average log number of CFU/mL of 8.18 ± 0.19 (*p*-value of 0.0022) and 8.10 ± 0.16 (*p*-value of 0.0022), respectively. The fixation plates loaded with 5% of GS completely prevented the attachment of *S. aureus* and killed all bacteria in the medium due to GS released. The numbers of bacteria, either attached to the fixation plates or present in the medium, did not differ between PCL and PCL-HA-HNT fixation plates, indicating that the incorporation of HA and HNT did not provide any antimicrobial activity to the plates (Figure 8A,B). Moreover, bacteria attached to the fixation plate were visualized by SEM. A high number of bacterial cells were found on the surface of PCL and PCL-HA-HNT fixation plates. In contrast, few bacterial cells were observed on the surface of the PCL-HA-HNT 2% GS plate, and no bacterial cells were seen on the surface of PCL-HA-HNT 5% GS (Figure 8C).

### 3.7. Ex Vivo Mouse Experiment

**Figure 9:**
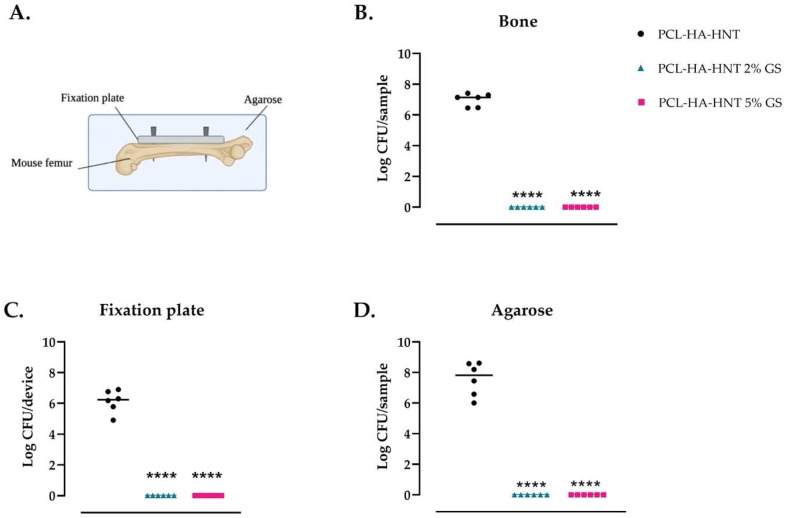
Antimicrobial activity of the fixation plates against *S. aureus* JAR 06.01.31 in an ex vivo mouse femur fixation plate infection model. PCL-HA-HNT plates, either non-loaded or loaded with 2 or 5% GS, were fixed to the femurs with 2 titanium pins and then inoculated with 1 μL containing 10^4^ CFU through a drilled hole in the center of the femur of (Ø 0.5 mm) Then, the inoculated femur with the fixed plate was placed in a container and covered with low melting agarose to mimic the surrounding tissue and incubated for 24 h. Schematic representation of the ex vivo model (**A**) and the quantification of bacterial colonies of *S. aureus* retrieved from the bones (**B**), the biomaterial (**C**) and agarose (**D**). Note: *n* = 6 per group. **** Indicates a *p*-value < 0.0001 versus the non-loaded plates.

In the ex vivo murine femur fixation plate infection model, the plates made of the composites loaded with 2 and 5% GS assay showed the complete killing of *S. aureus* in the bone, on the fixation plate and in the surrounding agarose after one day of incubation (Figure 9). Conversely, PCL-HA-HNT plates had an average log number of CFU of 6.99 ± 0.41 in the bone, 6.14 ± 0.73 on the plate and 7.57 ± 1.09 in the agarose (Figure 9).

### 3.8. In Vivo Antibacterial Activity of the 5% GS-Loaded PCL-HA-HNT Fixation Plates in a Mouse Bone Fixation Plate Infection Model

**Figure 10:**
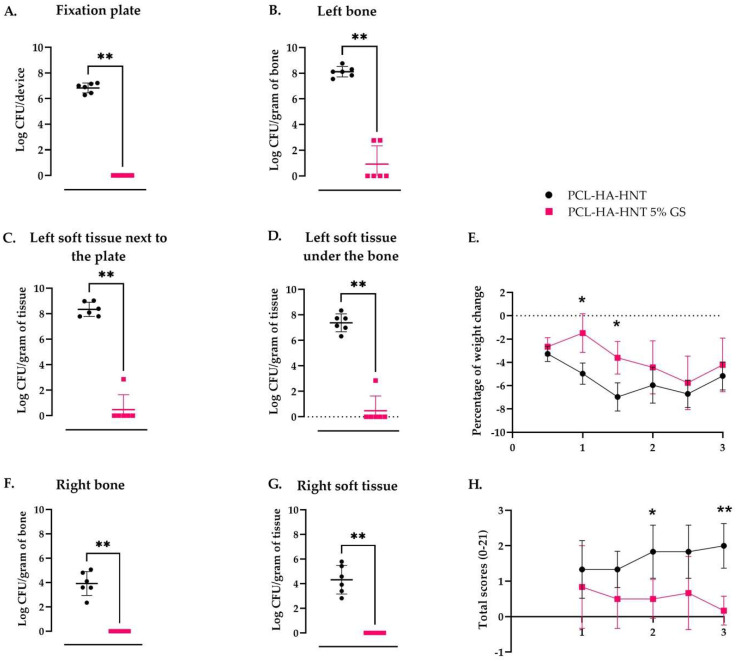
In vivo antimicobial activity in a murine fixation plate associated *S. aureus* bone infection of PCL-HA-HNT plates loaded with or without 5% GS on a fixation plate associated *S. aureus* bone infection in mice. Mouse femurs were fixed with a non-loaded PCL-HA-HNT plate or loaded with 5% of GS and then infected with 1 μL with 10^4^ CFU of *S. aureus* JAR 06.01.31 in a hole of 0.5 mm drilled in the center of the bone. After 3 days, the number of viable bacteria on the plate, in the bone (right and left), in the surrounding tissue next to the plate and in tissue under the bone were determined. Results are indicating the logarithmic quantitative bacterial growth per device (**A**), fixation plate or gram of bone (**B**), left bone; (**F**), right bone or tissue (**C**) left soft tissue next to the plate; (**D**), left soft tissue under the bone, (**G**), right soft tissue; (**E**), percentage of weight loss; (**H**), total scores. *n* = 6 mice per group Note: * Indicates a *p*-value of 0.01 to 0.05, ** indicates a *p*-value of 0.001 to 0.01.

To study the ability of PCL-HA-HNT fixation plate loaded with 5% GS to prevent infection by *S. aureus*, a mouse bone fixation plate infection model was used. The plate loaded with 5% GS was selected for its higher GS released and higher antimicrobial activity achieved in vitro compared to the plate loaded with 2% GS The performance of PCL-HA-HNT fixation plates loaded with 5% GS and non-loaded PCL-HA-HNT fixation plates was compared. After 3 days post-surgery, the plates, bones and soft tissue of all mice (*n =* 6 per group) with the non-loaded fixation plates were culture-positive, with an average log number of CFU of 7.12 ± 0.38 on the fixation plates, an average of log number of CFU/gram of 8.11 ± 0.41 in the left bone, 8.34 ± 0.56 in the soft tissue next to the plate and 7.37 ± 0.71 in the soft tissue under the bone. For all six mice with the plates loaded with 5% of GS, the fixation plates were culture-negative; of four mice the bone was culture-negative, and of five mice the soft tissue next to the plate and under the bone were culture-negative (Figure 10A–D). Infected mice with non-loaded PCL-HA-HNT plates showed an overall higher weight loss from day 1 to day 3 compared to the mice with the plates loaded with GS, particularly that was significantly different on day 1 post-infection (Figure 10E). Moreover, bacterial colonies were retrieved from the right leg (non-operated, non-inoculated) leg in the PCL-HA-HNT group showing an average log number of CFU/gram of 3.92 ± 0.98 in the bone and 4.32 ± 1.16 in the soft tissue (Figure 10F,G).

Moreover, the total welfare scoring indicated a higher level of discomfort for the mice with non-loaded plates compared to the mice with GS-loaded plates at each monitored time point. Differences were significant after 2- and 3-days post-surgery (Figure 10H).

## 4. Discussion

The principal strategy used in orthopedics to prevent or treat orthopedic infections is based on the local use of antibiotics such as GS, vancomycin or tobramycin impregnated polymethylmethacrylate (PMMA) cement or beads. PMMA bone cement loaded with antibiotics is used in the operating theater and placed as a coating for intramedullary nails or as spacers for treatment. Recently, a study incorporating PMMA cement loaded with GS and vancomycin applied on fixation plates showed a successful eradication of the infection in human patients [23]. Fixation plates coated with a cement impregnated with antibiotics are less invasive, easier to extract and can better release antibiotics to the surrounding tissue and bone than intramedullary nails [23]. However, PMMA cement is known to have poor release profiles that could lead to antimicrobial resistance eventually [24,25]. Moreover, they are made by hand mixing during surgery, making standardization of the process difficult. Moreover, as they are not biodegradable, which makes a second operation necessary for extraction, in case this is required.

In this study, we developed 3D printed PCL-HA-HNT fixation plates loaded with GS by FFF. First, filaments were produced via hot-melt extrusion (HME) and characterized. PCL melting point can be reached at around 60 °C; however, the melt viscosity may vary with and without HA and HNT. This has a direct impact both on the HME and 3D printing process [16,26,27]. During HME, there was no large variation in the extrusion temperature (around 10 °C change) because of the relatively large extrusion die. On the other hand, because of the small diameter of the nozzle used during 3D printing, an increase in the printing temperature was required. The printing temperature for PCL was 120 °C. An increase of 10 degrees was required when printing with PCL-HA-HNT compared with pure PCL. However, after adding the GS, an extra increase of 50 degrees was needed. This increase in temperature was key to reaching a suitable melt viscosity to have a successful extrusion through the printer’s nozzle. Additionally, any non-melted particles may increase the melt viscosity depending on their size. In our study, the HA particle size as described by the manufacturer was around 5 µm. Additionally, HNT were in the nanometer scale. Moreover, the GS particles had sizes up to 100 µm [9]. This may have been the cause for the need for a higher printing temperature of the composite to achieve a successful procedure. It is crucial to stay below the degradation temperature of GS and at the same time reach the proper melt viscosity during HME and 3D printing. It was confirmed that GS did not lose its antimicrobial activity after heating at the HME temperature. Moreover, the thermal properties of the produced filaments were investigated. There was a slight decrease in the crystallinity of PCL due to the presence of the particles of HA, HNT and GS which occupied the space within the polymeric matrix needed for crystal growth [28]. Fortunately, the mechanical properties (both tensile and flexure elastic module) were improved after adding HA and HNT. This increase can be due to interaction between PCL and HA and HNT [12] such as the hydrogen bonding between the hydroxyl groups on the surface of HNT and the carboxyl in PCL. Additionally, adding GS seems to cause a slight change to the mechanical properties compared to only adding HA and HNT.

The release kinetics of the two drug formulations, with the loading of 2% and 5% of GS, showed the same profile of a burst release of GS in the first 24 h that was followed by a sustained release of GS above the MBC for *S. aureus* during the following 13 days. This initial burst release was likely due to the presence of GS particles at the surface of the PCL-HA-HNT plates, while the sustained release later on was because of the water diffusion within the PCL matrix [9]. The in vitro adhesion assay of the non-loaded fixation plates or the plates loaded with 2 or 5% of GS showed a significant reduction in *S. aureus* colonies in the 2% GS plate and it’s medium and complete eradication of *S. aureus* on the plate loaded with 5% of GS and in its medium. In the ex vivo mouse femur fixation plate infection model, PCL-HA-HNT loaded with 2 and 5% of GS showed the complete killing of the *S. aureus* bacteria in the agarose, fixation plate and the bone. As our aim was to evaluate the ability of GS to be released from the PCL-HA-HNT plate, penetrate the infected bone and tissue and kill the bacteria, PCL-HA-HNT loaded with 5% GS was selected for in vivo evaluation. This plate was chosen for the in vivo mouse bone fixation plate infection model because it released a higher amount of GS in vitro (378.62 μg) and had shown a high antimicrobial efficacy both on the fixation plate and the medium in vitro. PCL-HA-HNT plate loaded with 5% of GS showed complete eradication of *S. aureus* in vivo in four out of six mice at 3 days post-surgery in the bone, soft tissue next to the plate and under the bone and fixation plate. Interestingly, the non-operated and non-inoculated leg of the mice was culture positive for the bone and the soft tissue in the mice group with PCL-HA-HNT with low numbers of bacteria, but not for the mice with GS-loaded plates. The culture positivity could have been caused by contamination during the collection of the samples. This is however unlikely, since the procedures to collect materials from the left leg were separated from those of the right, non-operated leg, and sterility precautions were carefully followed. Another explanation of the culture positivity may be the dissemination of the bacteria through internalization by macrophages, which can migrate through the bloodstream through the body, a mechanism also known as dissemination through “trojan horse” macrophages [4,18,29]. Since in mice with GS-loaded fixation plates no culture positivity of the right leg samples was found, GS apparently prevented this way of dissemination of the bacteria from the right leg, possibly by killing or “poisoning” the bacteria before they could be transferred to other sites of the body. This might be an additional positive effect of GS releasing fixation plates. More detailed studies should be performed to unravel whether this mechanism is operating here.

Previous studies incorporated GS in a poly (D, L)-lactic acid (PDLLA) coating of titanium intramedullary nails and studied the antimicrobial efficacy of the system in a rat bone implant-associated infection model [30,31]. Moreover, the release of GS was quantified in vivo from such PDLLA coatings of titanium intramedullary nails in a rat model showing a high concentration of GS in the bone at earlier time points (1 and 4 h) followed by a high concentration of GS in the kidneys at 1 and 3 days [7]. The local delivery of GS from a coating inside the bone impedes the distribution of GS into the surrounding tissue [32]. When delivered locally from the coating of a fixation plate, GS is expected to have better penetration of surrounding tissue because of the locally high concentration, such as released on the first day during the burst release from our fixation plates. Our quantitative results from the in vivo experiment indicate that GS must have distributed into the bone as well as into the surrounding tissue, as only a few colonies if any were retrieved from bone and tissue samples.

A GS-PDLLA coating of titanium successfully prevented the development of infection in vivo in a rat bone infection model [30]. Currently, this GS-PDLLA coating is used in the clinic on an intramedullary titanium nail for tibia fracture repair (Expert Tibia Nail PROTECT) [33,34]. Compared to this GS-PDLLA coating, our 3D printed biomaterial showed a higher and more sustained GS release. Moreover, our 3D printed system has the advantage of producing personalized geometries, shapes and drug loadings, allowing adaptation of the orthopedic device to the patient’s needs.

Mice have previously been used extensively for bone infection models to mimic trauma or an orthopedic infection with an intramedullary nail or a fixation plate [35]. In the bone-associated infection models that incorporate an internal fixation plate, such a plate is fixed to the bone with screws and a complete fracture was created [21,36,37] (reviewed in [35]). In our present study, the fixation plate was sutured to the bone instead of using screws, because the fixation plate was not designed for mechanical stability but as a model release system for antibiotic delivery, eventually to be applied as a coating to a mechanically stable fixation plate. Moreover, the infection was established by pipetting the inoculum into a pre-drilled hole in the bone instead of creating a complete fracture or osteotomy [21,36]. These adaptations to the model proved to be very effective and allowed us to study the efficacy of the release in vivo of the 3D printed plates.

In conclusion, PCL-HA-HNT-GS composite allowed the 3D printing of a release system for bone fixation plates or other devices which successfully prevented the development of *S. aureus* bone infection in vivo. The release profiles showed promising characteristics when compared with other commercially available formulations such as GS-PMMA. The technology thus has the potential for manufacturing effective antimicrobial devices for personalized applications.

## Figures and Tables

**Table 1:** Scoring system to monitor animal welfare [20,21].

Parameter	Scores
General behavior (posture and facial expression)	0/2/4
External appearance (eyes, fur, skin)	0/1/3
Movement	0/1/2/3
Faeces/Urine	0/2
Breathing	0/2/3
Wound healing	0/1/3
Grimace score	0/1/3

**Table 2:** DSC analyses results from produced filaments showing T_m_ and crystallinity of PCL (*n* = 3).

	PCL	PCL-HA-HNT	PCL-HA-HNT 2% GS	PCL-HA-HNT 5% GS
T_m_ (°C)	62.5 ± 0.4	61.0 ± 0.3	60.3 ± 0.7	60.5 ± 0.0
Crystallinity (%)	51.9 ± 0.2	50.1 ± 0.3	50.6 ± 0.6	50.9 ± 0.2
